# Phytic acid in green leaves of herbaceous plants—temporal variation *in situ* and response to different nitrogen/phosphorus fertilizing regimes

**DOI:** 10.1093/aobpla/plu048

**Published:** 2014-08-13

**Authors:** Hassan Hadi Alkarawi, Gerhard Zotz

**Affiliations:** 1 Functional Ecology Group, Institute of Biology and Environmental Sciences, University of Oldenburg, Box 2503, 26111 Oldenburg, Germany; 2 Foundation of Technical Education, Al-Musaib Technical College, Babylon, Iraq

**Keywords:** myo-Inositol hexakisphosphate, nutrients, phosphorus, phytate, relative growth rate (RGR), roots, storage

## Abstract

Phytic acid is the major storage compound for phosphorus (P) in plants. While accounting for up to 90 % in many seeds, usually only <10 % of total P is found in phytic acid in green leaves. This study follows up on the findings of a recent review of the occurrence of phytic acid in green leaves which revealed that (i) the current knowledge of phytic acid in leaves is mostly based on data from (fertilized) crop plants and (ii) the proportion of total P in phytic acid seems to *decrease* with improved P status in leaves in contrast to an *increase* in seeds and fruit. We studied five species of wild herbaceous plants in the field and under controlled conditions. Foliar P concentrations were much lower than those of the crops of earlier studies, but the proportion of P in phytic acid was similar, with little variation during the observation period. Both the field data and the experimental data showed a statistically indistinguishable negative correlation of phytic acid-P/total P and total P. In contrast to our expectation, this negative relationship was not related to differences in relative growth rates. We conclude that (i) our data of phytic acid concentrations in leaves of wild plants are in line with earlier observations on crops, and (ii) the trend towards lower proportions of phytic acid-P with increasing P status is probably a general phenomenon. Currently lacking a convincing explanation for the second observation, the role of phytic acid in foliar P metabolism is still unclear.

## Abstract (precis)

Phytic acid is the well-known principal storage compound of phosphorus (P) in fruits. Its metabolic role in other plant organs, e.g. green leaves is much less clear. Studying phytic acid in leaves of several herbaceous plant species, we obtained an expected (generally low foliar phytic acid concentrations) and an unexpected result: the proportion of P in phytic acid decreased when total P increased. We explored possible mechanisms behind this observation such as a link with plant growth rates, but did not find a convincing explanation for our surprising finding. Future studies should analyze all foliar P-species to resolve this puzzle.

## Introduction

Phytic acid or phytate, the free-acid form of myo-inositol hexakisphosphate (InsP6), is almost ubiquitous in eukaryotes. Its role in the storage of phosphorus (P) is well established, but there are other known functions, e.g. the storage of minerals such as K, Mg or Ca (review in Raboy 2003) or their involvement in a number of metabolic pathways at low concentrations (Munnik and Vermeer 2010). A large body of scientific work has focused on the occurrence of InsP6 in mature seeds, where it typically accounts for 60–80 % of total P (Greenwood *et al.* 1984; Batten and Wardlaw 1987; Raboy 2009). Generally low concentrations in leaves, on the other hand (e.g. deTurk 1933), did not suggest any major storage function of this compound in green foliage. This view has been challenged recently by the results of Winkler and Zotz (2009). Studying the uptake and allocation of P in foliage of fertilized epiphytic bromeliads, these authors found up to 20 % of total P in phytic acid (Winkler and Zotz 2009; U. Winkler, unpubl. res.).

In an effort to put these results into perspective, Alkarawi and Zotz (2014) reviewed the literature for information on phytic acid concentrations in green leaves of other plants. Phosphorus in phytic acid of 35 plant species accounted for 1–27 % of total P, with an average of 8 % and a median of 5 %. Although these values are much lower than those found in typical storage tissues of reproductive organs, the concentrations seem too high to be solely explained by the signalling functions of phytic acid. Moreover, the database proved to be highly biased towards crops. Few wild herbaceous plants have been studied in this regard, leaving the issue of the role of phytic acid in leaves of natural vegetation still unresolved.

Alkarawi and Zotz (2014) made another particularly surprising observation—although phytic acid increased with increasing total P, there was a ‘negative’ correlation between the ‘proportion’ of phytic acid and total P in *Manihot esculenta* leaves, although the opposite relationship is usually found in mature seeds and fruit (Eeckhout and Depaepe 1994). A positive relationship can be easily explained by the fact that phytic acid synthesis usually starts as soon as the supply of P exceeds the needs of basic plant metabolism when no other sinks for P are present: a positive correlation is thus inevitable (Bieleski 1973). A negative correlation is more difficult to explain. A constant ‘residual’ level of phytic acid in leaves would lead to the observed negative correlation, but there is no indication for such a relatively constant, residual amount of phytic acid in leaves (Alkarawi and Zotz 2014). Another possible explanation relates foliar P levels to plant growth. If low P concentrations in leaves were associated with slow plant growth (and consequently low sink strength) and higher P concentrations with fast growth (and high sink strength), the proportion of ‘inactive’ P found in phytic acid should decrease with increasing total P.

The present research set out to study phytic acid in leaves and other plant organs of five species of wild plants with a focus on one species, *Taraxacum officinale* (L.) Weber ex F.H.Wigg. We wanted to document *in situ* foliar concentrations of phytic acid in wild plants, but more importantly explore a possible link between plant growth and relative changes in foliar phytic acid concentration with data from the field and from a full-factorial experiment in a screenhouse.

## Methods

### Temporal changes in nitrogen (N), P and phytic acid contents *in situ*

#### Species and site description

We studied a total of five species with a focus on *T. officinale* (Asteraceae). This species and *Rumex acetosa* L. (Polygonaceae) are herbaceous perennials; a third species, *Alliaria petiolata* (M.Bieb.) Cavara & Grande (Brassicaceae), is a biennial; and *Galium aparine* var. agreste L. (Rubiaceae) and *Vicia angustifolia* L. (Fabaceae) are annual herbs. All sampled plants were naturally growing at three sites close to the campus of the Oldenburg University, in northwest Germany (53°9′0″N, 8°10′12″E).

#### Sampling and analyses

Between early April and late June 2012, we sampled one to three individuals per species (three per site in the case of *T. officinale*) about every other week. All plants were selected at the onset of the census to assure that plants were of relatively homogeneous initial appearance. After each harvest, we immediately determined total plant fresh weight (FW) and the FW of each compartment, i.e. leaves, roots, stem(s) and—when present—flowers and fruits. Dry weights (DW) of all plant parts were determined after drying at 100 °C for 24 h in a ventilated drying oven. Samples were ground using a ball mill (MM200, Retsch, Haan, Germany). Total N and carbon (C) concentrations were analysed using a Flash EA 1112 Series elemental analyzer (Thermo Electron Corporation, Delft, the Netherlands). Total P was determined colorimetrically using ammonium heptamolybdate (Chapman and Pratt 1961). Phosphorus in phytic acid was assayed calorimetrically with a phytic acid/total P assay kit (K-PHYT; Megazyme International, Wicklow, Ireland). This kit measures P released from a ground sample after treatment with phytase and alkaline phosphatase. Samples not treated with phytase allow the quantification of monophosphates not associated with phytic acid, which is necessary to avoid an overestimation of P in phytic acid. Since P comprises 28.2 % of phytic acid, multiplying phytic acid-P with the factor of 3.55 yields the amount of phytic acid. As suggested by the manufacturer, we routinely used oat samples supplied with the kit as a control. All samples of *T. officinale* were analysed with this kit, but only a subset of the samples of the other species.

For *T. officinale*, relative growth rates (RGR) preceding each sampling date were calculated following Evans (1972):

RGR=(lnW2−lnW1)/(t2−t1),

where *t*1 is the initial sampling date, *t*2 is the subsequent sampling date, *W*1 is the total plant DW at *t*1 (in g) and *W*2 is the total plant DW at *t*2. Sampling four times yielded three estimates of RGR.

### Growth and nutrient relations of *T. officinale* under different levels of N and P supply

Growth and plant nutrient status of *T. officinale* under different nutrient regimes was studied in a full-factorial experiment between 8 August and 3 October 2012 in the Botanical Garden of the University of Oldenburg. Seeds of *T. officinale* were germinated in 72 pots of soil mix (sand : peat : loam 40 : 40 : 20) with one seedling per pot (10 × 10 × 10 cm). Three different levels of N were combined with three levels of P, each fertilization treatment with eight replications. The N concentrations were 0.01, 0.14 and 1.3 mM, and the P concentrations were 0.01, 0.08 and 0.88 mM. Plants were irrigated with fertilizer solutions three times per week. For an estimate of RGR, we determined the average DW of 100 seeds, which was used as initial plant weight (*W*1), while *W*2 varied depending on the DW of plants at final harvest 8 weeks after the start of the experiment. For each plant, total FW, total DW, leaf DW, root DW and total leaf area were determined (using digital photographs and Adobe Photoshop^®^). Then, samples were ground using a ball mill (MM200), and N, C, P and phytic acid-P were determined as described above.

### Statistical analysis

All statistical tests were performed with the program R 2.15.0 (R Core Team 2012). Before applying parametric tests, we used appropriate tests to assure that assumptions such as normal distributions or homoscedasticity were met. Field data of *T. officinale* were obtained from three different sites. Since we found no site effect in an initial analysis of variance (ANOVA, *P* > 0.05), data of each sampling date were pooled in subsequent analyses. Statistically significant results of an ANOVA were followed up with Tukey's honest significant difference tests to identify significant between-group differences. Pearson's product-moment correlations were computed to analyse linear relationships between parameters. An analysis of covariance (ANCOVA) was performed for a comparison of the relationships of P in phytic acid and total P in leaves of field-grown and experimental plants. As critical significance level we consistently used *P* < 0.05.

## Results

### Seasonal changes in growth and nutrient contents

**Figure 1.**
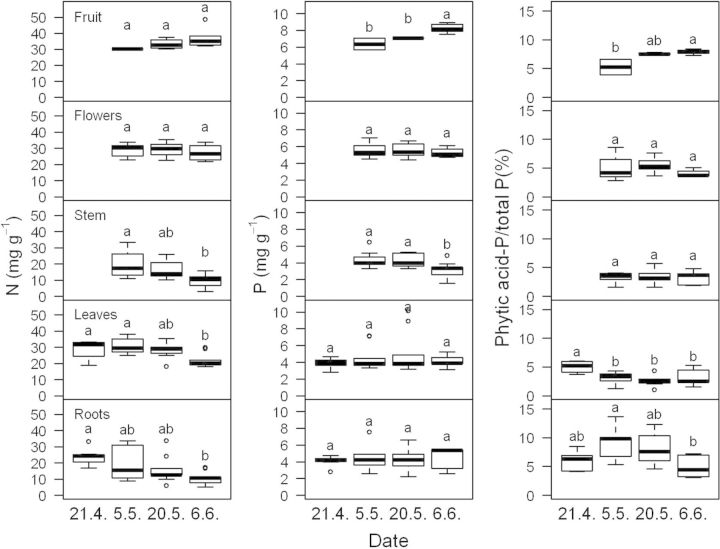
Concentrations of N (mg g^−1^) and P (mg g^−1^), and proportion of P in phytic acid and total P (%) in *T. officinale* plants. Nine entire plants separated into roots, leaves, stems, flowers and fruits (when present) were sampled every other week from 21 April 2012 to 6 June 2012. Different letters represent significant differences (Tukey's test following ANOVAs, *P* < 0.05).

During the study period, plant DW of *T. officinale* more than doubled from 2.6 ± 1.1 g (mean ± SD, *n* = 9) in April to 5.8 ± 1.9 g in June. Aboveground biomass more than tripled from 1.3 ± 0.6 to 4.2 ± 1.3 g in the same period. The concentrations of N decreased consistently in both roots and shoots during the study period, whereas P concentrations did not change with time (Fig. 1). The proportion of total P found in phytic acid, which averaged 3–5 % in leaves, was highest at the initial sampling date, with no further changes in the three subsequent sampling dates. A comparison with the temporal variation in the other four species did not indicate any consistent temporal trend in either P or phytic acid (Table 1). The proportion of P found in phytic acid was similar in *A. petiolata* with 3.5 ± 0.5 % (mean ± SD, *n* = 4 sampling dates) and more than twice as high in *G. aparine* with 12.2 ± 4.0 % (mean ± SD, *n* = 4 sampling dates). Phytic acid was also detected in all other studied plant organs of *T. officinale*, i.e. stems, flower heads and fruit. Surprisingly, phytic acid concentrations in fruits were also rather low accounting for <10 % of total P.

**Table 1.** Temporal changes in the mean concentrations of P and phytic acid in green leaves of selected herbaceous plant species.

Species	Family	Sampling date	*n*	Total P (mg g^−1^)	Phytic acid (mg g^−1^)	Phytin-P (mg g^−1^)	Phytin-P (% total P)
*A. petiolata*	Brassicaceae	21 April 2012	1	2.3	0.35	0.10	4.1
		5 May 2012	1	3.1	0.31	0.09	2.8
		20 May 2012	2	2.3	0.30	0.08	3.6
		6 June 2012	2	4.1	0.51	0.14	3.5
*G. aparine*	Rubiaceae	21 April 2012	1	2.7	1.24	0.35	12.9
		5 May 2012	1	3.2	0.91	0.26	8.1
		20 May 2012	3	3.0	1.13	0.32	10.6
		6 June 2012	3	2.1	1.29	0.37	17.5
*R. acetosa*	Polygonaceae	21 April 2012	1	3.3	0.62	0.17	5.2
		5 May 2012	1	3.2	0.43	0.12	3.8
		20 May 2012	2	2.9	0.31	0.09	3.1
		6 June 2012	2	2.2	0.52	0.15	6.6
*T. officinale*	Asteraceae	21 April 2012	9	3.9	0.68	0.19	5.03
		5 May 2012	9	4.5	0.47	0.14	3.13
		20 May 2012	9	5.2	0.45	0.13	2.73
		6 June 2012	9	4.0	0.46	0.13	3.37
*V. sativa*	Fabaceae	21 April 2012	1	1.9	0.26	0.07	3.8
		5 May 2012	1	2.0	0.41	0.12	5.9
		20 May 2012	3	1.5	0.32	0.09	6.0
		6 June 2012	2	1.5	0.34	0.10	6.4

**Figure 2.**
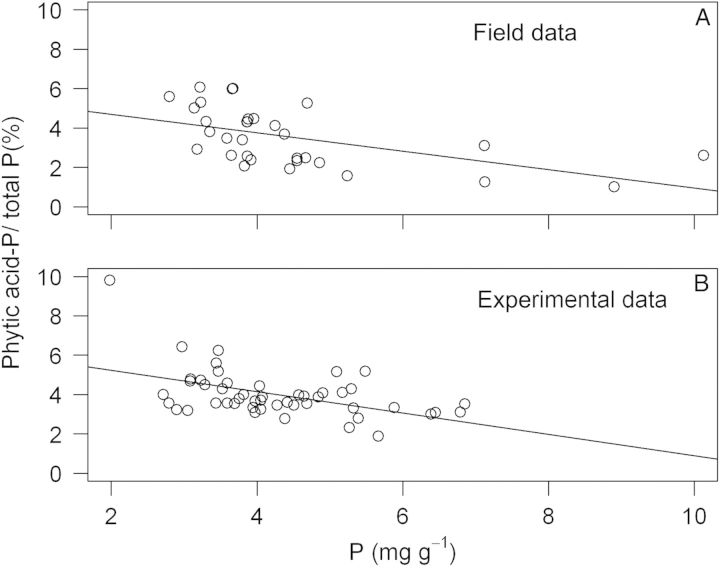
The proportion of P in phytic acid and total P (%) as a function of total P in leaves of *T. officinale*. The upper panel (A) shows data from field samples that were collected between 21 April and 6 June 2012. The lower panel (B) shows data from juveniles, which were subjected to nine different treatments (three N and three P levels) in an experiment lasting 8 weeks. The regression lines are phytic acid-P/total P = 5.62−0.47, total P (field; *R*^2^ = 0.28, *P* < 0.001) and phytic acid-P/total P = 6.32−0.54, total P (screenhouse experiment; *R*^2^ = 0.24, *P* < 0.001). Slope and intercept of the two regression lines do not differ (Table 3).

The average relative growth rate of *T. officinale* decreased from 53 ± 19 mg g^−1^ day^−1^ (mean ± SD, *n* = 9) between the first and second sampling dates over 26 ± 12 to −13 ± 3 mg g^−1^ day^−1^ between the last two sampling dates. This consistent reduction in RGR was not accompanied by a similar change in the proportion of P in phytic acid (Fig. 1). On the other hand, the correlation of the percentage of P in phytic acid and total P in leaves, including all individual data points obtained for *T. officinale*, was significantly negative (Fig. 2A, *R*^2^ = 0.28, *P* < 0.001, *n* = 36). The same was true for roots (*R*^2^ = 0.18, *P* < 0.01, *n* = 36).

### Growth and nutrient relations of *T. officinale* under different levels of N and P supply

**Figure 3.**
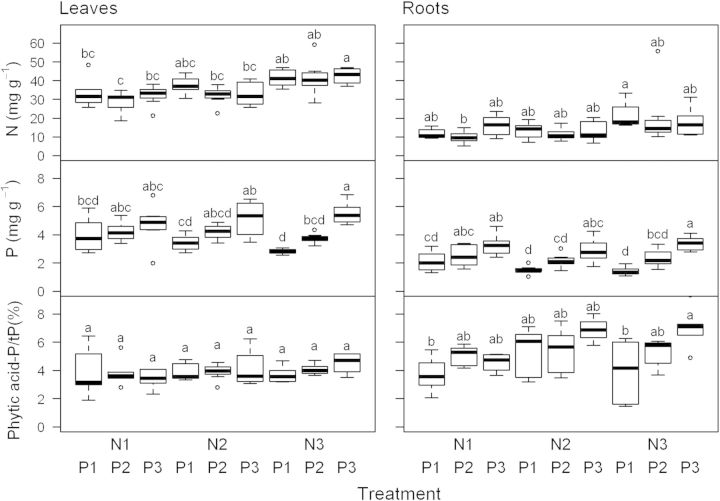
Concentrations of N (mg g^−1^) and P (mg g^−1^), and the proportion of P in phytic acid and total P (%) in leaves and roots of *T. officinale* plants as a function of nine different combinations of N and P supply. N1–N3: increasing levels of N, P1–P3: increasing levels of P. Different letters represent significant differences (Tukey's test following one-way ANOVAs). Sample sizes are *n* = 8 for N and P and *n* = 3 for phytic acid.

The RGR of *T. officinale* over the 8 weeks of the fertilizer experiment were about 2-fold higher than the highest rates observed in the field, ranging from 75–120 mg g^−1^ day^−1^. Different supply of N, but not of P, had a significant effect on RGR (Table 2). Increasing levels of N and P supply led to higher foliar N and P concentrations (two-way ANOVAs, *P* < 0.05), although this effect was much more consistent and pronounced for P (Fig. 3). Foliar nutrient concentrations, in turn, correlated significantly with RGR only in the case of N (Pearson's product-moment correlation, *R*^2^ = 0.09, *P* = 0.04), not in the case of P (*R*^2^ = 0.01, *P* = 0.45). Remarkably, the much larger variation in foliar P concentrations compared with the field data did still not result in any change in the proportion of P found in phytic acid. It remained at ∼4 % in all treatment combinations (Fig. 3). Analysing the data with a regression analysis yielded again a significant negative correlation of the proportion of P in phytic acid and total P (Fig. 2B, *R*^2^ = 0.24, *P* < 0.001, *n* = 48). Combining both data sets from the field and the screenhouse experiment in a single ANCOVA revealed that the relationships of P in phytic acid and total P in leaves were statistically indistinguishable (Table 3, Fig. 2).

**Table 2.** Two-way ANOVA: effect of fertilization treatments (N = nitrogen and P = phosphorus) on relative growth rate in *T. officinale*.

Factor	d.f.	*F*	*P*-value
P	2	0.227	0.80
N	2	14.968	<0.001
P × N	4	1.122	0.35
Residuals	63	0.005

**Table 3.** Analysis of covariance of the effects of foliar P concentration and study system (field vs. experiment) on the proportion of P stored in phytic acid in *T. officinale*.

Factor	d.f.	*F*	*P*-value
P	1	29.2	<0.001
Study system	1	2.3	0.13
P × SS	1	0.15	0.70
Residuals	81	0.005

Compared with leaves, roots had much lower concentrations of both N (∼40 %) and P (∼50 %), but the relative treatment effects on the concentrations of N and P in these underground organs were similar to those found in foliage (Fig. 3). Treatment-related variations in phytic acid concentrations were much more pronounced in roots, but the proportion of phytic acid and total P was not correlated with total P (Pearson's product-moment correlation, *R*^2^ = 0.01, *P* = 0.5).

## Discussion

This study had two main objectives: (i) because of the strong bias towards crops in our current database of phytic acid in leaves we wanted to obtain data for wild plants and (ii) investigate the relationship of phytic acid and total P as a function of P supply and plant growth rates. The average P concentration in the crops reviewed by Alkarawi and Zotz (2014) was almost three times higher than that in the five field-grown wild species of the present study (8 ± 4 vs. 2.9 vs. 1.0 mg P g^−1^ DW, Alkarawi and Zotz 2014, App. 2; this paper, Table 2), but the proportion of P in phytic acid was similar (7.5 ± 5.0 vs. 5.9 vs. 3.7 %, Alkarawi and Zotz 2014, App. 2; this paper, Table 2). This supports the generality of our previous conclusion: phytic acid is ubiquitous in leaf tissue, but accounts for a rather small proportion of total P.

The metabolic role of phytic acid in leaves is still puzzling. Apart from being the major storage compound for P in seeds and fruit, a number of additional functions have been proposed for InsP6. These range from a ubiquitous involvement in cellular signal transduction and regulation in all eukaryotic cells (Lee *et al.* 2007; Nagy *et al.* 2009) at low concentrations to a possible, defensive function against insect herbivores in some plant species (Green *et al.* 2001). Other functions are reviewed in Raboy (2003). If primarily functioning as a storage compound similar to the situation in seeds one would expect a positive correlation of the proportion of phytic acid and total P (Eeckhout and Depaepe 1994); phytic acid synthesis starts when the supply of P exceeds the needs of basic plant metabolism. In contrast, although the absolute concentrations of phytic acid increased with total P in leaves, the relative proportions showed a significant and consistent decrease, both in mature plants in the field and in young seedlings in the screenhouse (Fig. 2, Table 3). Plant growth could provide an alternative sink for foliar P, which would also preclude local storage, because excess P is exported to other actively growing tissues, e.g. new leaves, flowers or fruit. However, we did not find a correlation between RGR and the proportional levels of phytic acid-P. Taken together, our findings are not compatible with the notion that phytic acid is an important temporary storage compound for P in leaves of the studied forbs. Unfortunately, we lack information on the changes in the concentrations of the other P species (e.g. orthophosphates, nucleic acids, phospholipids) with increases in total P. To understand P metabolism in leaves it will be necessary in future studies to quantify not only total P and phytic acid, but also all of these compounds containing P (compare Noack *et al.* 2012).

The low proportions of P in phytic acid in plant leaves render the finding of considerably higher levels of phytic acid in foliage of epiphytic bromeliads (>20 % of total P; Winkler and Zotz 2009; U. Winkler, unpubl. res.) even more interesting. Bromeliad leaves are already unusual in the plant kingdom. Besides their function as photosynthetic organs they take over, partially or entirely, the function of roots by absorbing water and nutrients with their foliar trichomes (Benzing 2000). The high levels of phytic acid in the studied bromeliads suggest that their leaves have also a major storage function, contrasting with relatively short-lived leaves of the present investigation. Such a role of phytic acid may not be restricted to epiphytes but also be found in other plant groups, in which long-lived foliage accounts for the major proportion of biomass such as leaf-succulent terrestrial genera, e.g. *Agave* or *Aloe*. Since the current understanding of phytic acid metabolism indicates that high levels of phytic acid are mostly associated with P storage (Schachtman *et al.* 1998; Munnik and Vermeer 2010), determination of foliar phytic acid levels could provide a convenient way to evaluate the P-nutrient status of vegetative tissue of such plants in the wild.

## Conclusions

The enigmatic finding of a negative correlation of the proportion of phytic acid-P and total foliar P in a recent review was based on combined data from several studies and could well have been fortuitous. Our new results provide support for the generality of this relationship, but do not resolve the underlying mechanism because our hypothesis, a link to plant growth, was not supported. The role of phytic acid in green leaves is still unclear—we suggest that this role may differ in plants varying in leaf longevity and/or leaf mass ratios.

## Sources of Funding

This study is part of the doctoral thesis of the first author. A stipend from Tabadul, the Iraqi-German Scholarship programme (DAAD), is gratefully acknowledged.

## Contributions by the Authors

H.H.A. and G.Z. conceived the study design. H.H.A. performed the experiment and the data analysis. H.H.A. and G.Z. wrote the paper.

## Conflicts of Interest Statement

None declared.
